# Assessing the Utility of Thermodynamic Features for microRNA Target Prediction under Relaxed Seed and No Conservation Requirements

**DOI:** 10.1371/journal.pone.0020622

**Published:** 2011-06-06

**Authors:** Parawee Lekprasert, Michael Mayhew, Uwe Ohler

**Affiliations:** 1 Institute for Genome Sciences and Policy, Duke University, Durham, North Carolina, United States of America; 2 Program in Computational Biology and Bioinformatics, Duke University, Durham, North Carolina, United States of America; 3 Departments of Biostatistics and Bioinformatics, and Computer Science, Duke University, Durham, North Carolina, United States of America; University of Edinburgh, United Kingdom

## Abstract

**Background:**

Many computational microRNA target prediction tools are focused on several key features, including complementarity to 5′seed of miRNAs and evolutionary conservation. While these features allow for successful target identification, not all miRNA target sites are conserved and adhere to canonical seed complementarity. Several studies have propagated the use of energy features of mRNA:miRNA duplexes as an alternative feature. However, different independent evaluations reported conflicting results on the reliability of energy-based predictions. Here, we reassess the usefulness of energy features for mammalian target prediction, aiming to relax or eliminate the need for perfect seed matches and conservation requirement.

**Methodology/Principal Findings:**

We detect significant differences of energy features at experimentally supported human miRNA target sites and at genome-wide sites of AGO protein interaction. This trend is confirmed on datasets that assay the effect of miRNAs on mRNA and protein expression changes, and a simple linear regression model leads to significant correlation of predicted versus observed expression change. Compared to 6-mer seed matches as baseline, application of our energy-based model leads to ∼3–5-fold enrichment on highly down-regulated targets, and allows for prediction of strictly imperfect targets with enrichment above baseline.

**Conclusions/Significance:**

In conclusion, our results indicate significant promise for energy-based miRNA target prediction that includes a broader range of targets without having to use conservation or impose stringent seed match rules.

## Introduction

MicroRNAs (miRNAs) are small, non-coding RNAs that have important roles in the post-transcriptional gene regulation in animals and plants, and are involved in a wide variety of cellular processes [1], [2]. As part of the RNA-induced silencing complex (RISC), miRNAs regulate gene expression through different mechanisms including destabilizing transcripts, promoting transcript degradation, and/or inhibiting translation [3]. While many miRNAs have been identified, until recently a relatively small portion of targets had been experimentally validated due to the low throughput manner that is generally accompanied with biological validation. Recent developments have enabled large-scaled identification of a direct interaction between miRNA and mRNA; however, these methods are still early in development, and typically cover an ensemble of active miRNAs rather than a single gene [4]–[7].

At this point, computational approaches are still the driving force in miRNA target prediction, and numerous tools have been developed to assist identification of miRNA targets. These tools can reduce the number of likely targets to a more manageable number for experimental validation. However, creating accurate target prediction tools has been an ongoing challenge. Several studies have shown that predicted target sets differ among target prediction tools [8], [9]. Some of the non-overlapping predicted targets may be a result of different 3′UTR sequences used, yet even when using the same sequence set for prediction, a large portion of targets predicted by different tools still do not overlap [10].

Multiple features have been shown to be informative for miRNA target prediction. Most of the algorithms make strong assumptions on the type of matches to the target sequence, in particular to the so-called seed region, which spans the first eight nucleotides of the miRNA [11], [12]. Target sites with perfect complementarity to position 2–7 or 2–8 are typically called canonical sites. Besides Watson-Crick base pairing, G-U base pairs in miRNA:target duplexes are counted as canonical match in some target prediction tools [8], [13], as is the presence of an adenine base across from position 1 of miRNA [12]. The second commonly used key feature in target prediction tools is evolutionary conservation; it provides a strong signal as many functional miRNA targets are conserved across species. Additional “context” features include, among others, AU content around the target site, and relative target site location in the 3′UTR [14], [15].

However, there are limitations to these key features in predicting miRNA targets. Requiring perfect complementarity to the 5′ seed region of a miRNA will leave out target candidates with imperfect seed match, such as those reported in [16]–[18]. A recent study reported centered sites, which lack perfect 5′ seed pairing but instead have contiguous base pairs from position 4 or 5 to position 14 or 15 [19]. Furthermore, miRNA targets that are not widely conserved will be missed if evolutionary conservation is required. A seed-relaxed approach is especially crucial in the context of viral miRNAs, which are used to regulate their own as well as their host's genes [20]; conservation-based target prediction is not applicable here as viruses evolve too fast and are typically highly adapted to a specific host [21]. Additionally, some viral miRNAs with extensive sequence similarity to host miRNAs have been shown to target genes differentially [22], [23], and traditional seed-based predictors will not be able to predict differences in genes targeted by either miRNA.

As an alternative approach, different studies have shown evidence of thermodynamic properties as signals for functional miRNA or siRNA targeting. The underlying idea of using thermodynamic properties is that gene regulation by miRNAs involves a direct binding between a miRNA and its mRNA target. This binding may be considered in terms of thermodynamics as a process where free energy changes occur via formation of a duplex between miRNA and mRNA, and such changes may help identify miRNA targets. Zhao et al. observed that miRNA target sites tend to reside in an unstable region, and tend to lack stabilizing elements, namely long stems [24]. Tafer and colleagues showed that target site accessibility improves predictions of highly efficient siRNAs [25]. In complementary work, several groups demonstrated that different context sequences around the same binding site affect the repression levels [9], [26].

The free energy change involved with mRNA:miRNA duplex formation may thus serve as a key predictor for miRNA targets. Continuous-valued thermodynamic features may also allow prediction of actual levels of suppression caused by miRNAs instead of a binary yes/no decision. Early on, thermodynamic properties have been used in some forms to predict miRNA targets, but tools vary greatly in terms of energy computation and its incorporation into a prediction model. The majority of these tools focus on one energy feature, hybridization energy, only [11], [13], [27]–[30]. Many of them only use energy as a filter for putative target sites, and are still largely dependent on seed match or conservation [11], [27], [28].

More recently, integrated thermodynamic features for miRNA target identification demonstrated the effectiveness of combining target accessibility and duplex stability [9], [26]. In addition, using data from pull-down experiments of miRNAs in the RISC, Hammell et al. showed that total free energy change and target accessibility yielded enrichments in miRISC-enriched transcripts [8]. However, these studies focused on model organisms with more compact genomes and comparatively short 3′UTRs (fly and worm), and several independent genome-wide studies on more complex human datasets concluded that the accuracy of at least some algorithms was not on practically relevant levels and did not significantly exceed scans for canonical seed matches [31], [32]. Besides the issue of differences between organisms, genomic predictions generally still required candidate sites to contain perfect seed match of length 6, or seed match of length 7 or 8 with one G-U base pair, and therefore did not specifically address the potential benefit of energy-based models to address the issue of imperfect sites [9].

These conflicting findings prompted us to independently reassess the usefulness of energy features for mammalian miRNA target prediction. We systematically evaluate the contribution of different energy features and seed requirements on known curated human target sites as well as recent genome-wide maps of Argonaute (AGO) family member binding sites, which provide global measurements of RISC and thus miRNA targeting. Then, we propose a simple linear prediction model and evaluate it based on genome-wide data on mRNA and protein expression changes induced by human and viral miRNAs. Our results show that it is possible to deliver energy-based target prediction that exceeds the performance of baseline seed match searches, even on strictly imperfect sites. Our results compare well against previous approaches, and indicate the potential for energy-based features on the way to develop flexible and tractable prediction models that can be used on a broader range of miRNA target predictions, including non-conserved and imperfect sites.

## Methods

Our approach is inspired by the previously proposed model that mRNA:miRNA duplex formation occurs in a stepwise manner [9], [26]. First, a portion of the mRNA where a target site resides has to become locally accessible to a targeting miRNA. The energy required to open up the local mRNA secondary structure around the target site is designated as the disruption energy, ΔG_open_. The second step is the binding of the miRNA to the open target site, and the free energy change in this binding step is called the hybridization energy, ΔG_H_. The total free energy change of the entire duplex formation (ΔΔG) is the difference between the hybridization energy and the disruption energy.

### Match site identification and energy computation

In an energy-based model, any position within a 3′UTR is a potential target site, albeit at different affinities. For practical reasons, we computed energy values at candidate target sites in the 3′UTR that contained a consecutive perfect 4-mer match within the canonical seed region to the miRNA (position 2–8). This minimum match length was motivated by Long et al. [26], where it was proposed that duplex formation requires a minimum nucleus of four nucleotides in length; however, different from that approach, we restricted this nucleus to the miRNA seed region. For hybridization energy (ΔG_H_) computation, we extracted the flanking sequence up to twice the length of the remaining miRNA portion at each side of the 4-mer match. The flanking sequence can be a part of a coding region of the mRNA. While interactions may potentially occur across a larger region, using longer context will result in mRNA structures with increasing internal base pairing, which does not reflect the energy changes occurring in the functional binding between miRNA and mRNA. We then used RNAcofold in the Vienna package to compute the free energy change during hybridization, and we required that the bases in the 4-mer match region were paired [33], [34]. In case of any unpaired bases in this region or internal base-pairing within the molecule, we used RNAeval in the Vienna package to recompute the energy value for the modified structure (paired at 4-mer, with the internal pairing removed) [34].

Since RNA secondary structure computation is computationally intensive, we computed the disruption energy (ΔG_open_) locally around possible candidate sites to be able to handle the frequently long mammalian 3′UTRs, akin to previous approaches [25]. We used RNAplfold in the Vienna package to compute unpaired probabilities P_unpaired_ of a local window with the parameter setting as follows: the window size of each local structure, W = 80; the maximum distance allowed between paired bases, L = 40; and the open region size, u = 20 [35], [36]. To integrate energy at both steps of the duplex formation, we converted the average accessibility probability across all sliding windows over the match site to the disruption energy value. Since the expectation value of natural log is not equal to the natural log of the expectation value, we extracted a sequence of exactly the length of the window size (W) so that the program reported the exact open probability of this single window. Then we converted this open probability value to the disruption energy value as follows: ΔG_open_ = RT ln (P_unpaired_). We repeated these steps over all possible sliding windows over the match site. The final ΔG_open_ value is the average of all ΔG_open_ values for each sliding window. The total free energy change (ΔΔG) is the difference between the hybridization energy (ΔG_H_) and the disruption energy (ΔG_open_): ΔΔG = ΔG_H_−ΔG_open_.

After computing these energy values, initial 4-mer match sites were screened for overlaps in cases when multiple overlapping 4-mers matched to the same region. We chose the site with the best ΔΔG value to represent these overlapping 4-mer matches. We validated our approach on example data, where the correlation between total free energy change and miRNA-induced repression level was examined and experimentally validated for a number of examples from *Drosophila* [9], using different RNA (co-)folding tools. The target set included sites in *hid* (targeted by *bantam*), *grim* (miR-2), and *rpr* (miR-2) UTR within wildtype or modified context sequence flanking the target site. Figure S1 shows that our method yielded similar correlation between ΔΔG values and the normalized luciferase ratios, compared with the original study [9].

#### Seed type assignment and filters

Any energy-based predicted sites contained at least a perfect 4-mer complementary match to the seed region from position 2–8. We evaluated a seed type of such sites based on maximal complement; the categories included 8 consecutive base pairs from position 1 to 8 (8-mer), 5 to 7 consecutive base pairs within positions 2 to 8 (5-mer, 6-mer, and 7-mer), 7-mer plus adenine across the first nucleotide of miRNA (7-mer-A), and 5 to 7 non-consecutive base pairs or an adenine across miRNA position 1 (5-in-8, 6-in-8, and 7-in-8). G-U base pairs were counted as mismatches in the initial match site identification and the seed type assignment.

We used seed match types as filters to define more/less stringent prediction sets. With a 6-mer filter, only the match sites that have at least 6 consecutive Watson-Crick base pairs to the miRNA (8-mer, 7-mer-A, 7-mer, 6-mer sites) were used. With a 6-in-8 filter, we only used the match sites that have at least 6 base pairs to the miRNA (an adenine across miRNA position 1 counted as a base pair for imperfect sites). This means a 6-in-8 filter allows for 8-mer, 7-mer-A, 7-mer, 6-mer, 7-in-8, and 6-in-8 match sites as defined above.

### Evaluation of energy contribution at experimentally supported human miRNA target sites

We evaluated energy features on known target sites of human miRNAs as reported in Tarbase version 4.0 [37]. For the positive set, we used all 112 miRNA-mRNA target pairs for which we could obtain an accurate mRNA sequence at the reported site from the UCSC genome annotation (hg18) [38]. For the control set, we randomly selected match sites of dinucleotide-shuffled miRNA to randomly assigned Tarbase UTR. To create a shuffled miRNA, the starting nucleotide was selected based on the nucleotide frequencies of the Tarbase miRNA. We then used a first-order Markov chain model to build up the rest of the sequence. Remaining nucleotides, which could not be incorporated while obeying first-order dependencies, were then randomly inserted if the initial successfully first-order sites exceeded 85% of the length of the miRNA. We filtered out any shuffled miRNAs whose seed sequence overlapped with any known miRNA seeds or poly-A motifs. We generated 10 shuffled miRNAs per Tarbase miRNA-mRNA target pair, with 10 randomly selected target Tarbase UTRs for each shuffled miRNA.

We compared cumulative density distribution of the energy values between Tarbase sites and control sites, and used Wilcoxon rank sum test to determine the significance of the differences between the distributions. To evaluate signals from energy features, we plotted the receiver operating characteristic (ROC) curves (true positive rates versus false positive rates over varying energy cutoffs) to distinguish between Tarbase sites and control sites, and computed the area under curve (AUC) values.

### Genome-wide evaluation of energy contribution at site level

For a genome-wide evaluation of features, we assessed the energy contribution in distinguishing sites with evidence of direct interaction by AGO proteins in HEK-293 cells as determined by the PAR-CLIP method, which involves cross-linking of proteins and mRNAs followed by deep sequencing of bound mRNA fragments [6]. Crosslinked-Centered Regions (CCRs) are 41 nucleotides long and centered at the site of highest evidence of direct interaction between an mRNA and an AGO complex within an initial cluster of reads. Even though CCRs were reported outside 3′UTRs as well, we used only the CCRs that are correctly mapped within human 3′UTRs in ENSEMBL47 [39] in order to be consistent with our studies on other datasets. All available transcript isoforms of a gene were used to search for matches in order to cover all possible target sites, including sites in alternative exons, but we only counted the same site within multiple isoforms once, unless differences in the local context sequence changed the energy values.

The PAR-CLIP experiments identified interactions between AGO proteins and mRNAs, but not directly for a specific miRNA. To create sets of confident miRNA-CCR pairs, we used the top 20 highly expressed miRNAs which accounted for a large fraction of possible target sites from the experiment (cf. [6]), and then evaluated CCRs for seed matches to these miRNAs only. In addition, since our method searches for matches to 5′end of a miRNA, we cannot exclude possible targeting by other miRNAs with similar seed sequence to these 20 miRNAs. Therefore, we included additional miRNAs with the same 5′end sequence (position 1 to 7, or 2 to 8) to these 20 miRNAs, and combined them all into 12 non-redundant miRNA groups for evaluation (Table S1).

We designated match sites of at least length 6 that fell within a CCR as positive sites; here we only used CCRs that have one match location to seed sequences of our miRNA set, and have sequence read numbers of at least 20. While this approach was carefully designed to include a large number of real target sites, it does not exclude the possibility that the CCR was targeted by a different miRNA, especially by ones outside the list of top 20 miRNAs, or that CCRs missed the real or best possible target location. Negative set members are 6-mer (or better) matches in 3′UTRs of expressed genes in HEK-293 cells (according to [40]) that lie outside the full CCR set. We plotted the ROC curve varying the energy cutoff for these positive and negative sets.

### Genome-wide evaluation at the UTR level

#### Dataset of miRNA-induced genome-wide expression change

The majority of genomic data on the effects of miRNAs assesses changes at the whole transcript level. We primarily used data from one study which compared miRNA-induced expression changes at the mRNA level (by microarrays) with those at the protein level (by pSILAC) for five human miRNAs — miR-1, miR-16, miR-30a, miR-155, and let-7b [32]. For mRNA expression change, we chose the values from microarray experiments measured 32 hours after transfection as they showed higher correlation to the change in protein production level than a sample obtained after 8 hours (cf. [32]). The expression change values here are log_2_ values of the ratio between signal in the presence of the miRNA and signal in the absence of the miRNA. We used the gene sequences that corresponded to human Refseq database version 26. Transcript variant coordinates from ENSEMBL 47 [39] were screened for the longest sequence among overlaps on the same strand. An annotated stop codon was required, and sequences were retrieved from the UCSC genome browser database (hg18) [38]. In our analyses, we only used the miRNA-gene pairs for which expression change were measured at both mRNA and protein levels. Across all five experiments, this totaled in 14,160 miRNA-gene pairs.

#### Training of linear prediction models

We evaluated different energy features, including three energy types at multiple sites, and the energy sum over all match sites in the 3′UTR. We built a linear regression model to predict expression change based on multiple energy features (e.g. individual and sum over all sites). The 3′UTR length was also included in the feature set, as longer 3′UTRs have increased chances for putative binding site matches. We used the lm() function in the R package to model parameters (i.e. feature weights), and evaluated the model on pooled expression data showing negative log_2_ fold change in a five-fold cross-validation setting. The Spearman rank correlation test was used to determine correlation between model-predicted score and observed log_2_ fold change. The expression change data at mRNA and protein levels were evaluated separately.

#### Enrichment analysis

To evaluate the performance for de-novo target prediction, we ranked the genes by their model-predicted scores and computed the enrichment of bona fide targets in top-scoring gene sets of increasing size (50 genes increment). Bona fide targets were defined as genes with observed log_2_ fold change less than or equal to a cutoff. The enrichment was defined as the ratio between the fraction of bona fide targets in our predicted set and the fraction of total bona fide targets in the full gene set. An enrichment value greater than one thus indicated that the predicted set contained a higher number of down-regulated genes than expected at random.

#### Performance comparison to PITA

We compared the performance of our method to PITA, a miRNA target prediction tool that uses energy values of the duplex formation [9]. Since PITA searches for match sites of length 6 or longer, we trained and evaluated our prediction model on 3′UTRs with at least one 6-mer (or better) site. Here, we limited our gene set to the overlap between the genes that we used from Selbach et al. [32] and PITA predictions (PITA Targets ALL catalog at the gene level, version 6) in order to compare the enrichment of highly down-regulated genes.

#### Comparison to context score

We compared the enrichment of bona fide targets in the top predicted sets based on our model versus context scores reported in TargetScanHuman release 5.1 [12], [14]. Context scores for candidate sites were computed based on site type, pairing at 3′ end of miRNA, local AU content, and position in the UTR [14]. We used the combined single-genome context scores of all sites in a 3′UTR (conserved and non-conserved) to rank genes for the enrichment analysis. As in the comparison to PITA, we used the regression models that were trained on 6-mer or better sites. The evaluation set contained the overlap between the TargetScanHuman UTR database, and our UTR set with at least one 6-mer (or better) site.

#### Evaluation of energy-based prediction model on independent expression change data

To evaluate our prediction model on a completely different dataset, we used mRNA expression change data obtained after transfection of miR-K12-11 in human B-cell line BJAB at physiological level [22]. The microarrays may contain multiple probes to the same 3′UTR in the experiment, and we consistently used the lowest log_2_ fold change (i.e. log_2_[(signal in the presence of the miRNA)/(signal in the absence of the miRNA)] ) among all probes for the UTR. We retrieved the sequences in the same way as for the Selbach et al. set above [32], yielding a total of 10,966 3′UTRs with associated expression values on this microarray. A subset of 9,379 3′UTRs that contained at least one 6-in-8 (or better) site was used in our enrichment analysis.

#### Software Availability

The Perl program to compute predicted miRNA-induced expression change of the gene according to our models is available at http://www.genome.duke.edu/labs/ohler/research/miRNAs/targetThermo/.

## Results

We evaluated contributions of energy features to miRNA target prediction on two different types of datasets: individual sites as annotated by human experts from the literature, or as predicted based on genome-wide RNA binding profiles, and miRNA-induced expression changes at the whole gene level. In the first case, we can directly address the contribution of individual energy features, whereas in the second case, multiple sites in 3′UTRs have to be combined into a single score.

### Known human target sites exhibit both significantly lower hybridization energy and higher disruption energy

We began the evaluation of energy features for target prediction on a curated set of experimentally supported human miRNA targets collected in Tarbase version 4.0 [37]. Tarbase provides a location of the target site in the gene, which we used as a positive site in our analysis. As there are insufficient negative sites that are known not to be targeted by any miRNA, we created 10 dinucleotide-shuffled miRNAs for each Tarbase-reported miRNA, and randomly assigned 10 Tarbase 3′UTRs to each shuffled miRNA to create randomized controls. We searched for match sites in these control miRNA-UTR pairs, and randomly selected one match site per UTR as a control site for comparison. This resulted in a stringent control set, as it only contained UTRs that were known to be targeted by miRNAs in Tarbase, and was as such different from a genome-wide randomly selected background.

**Figure 1:**
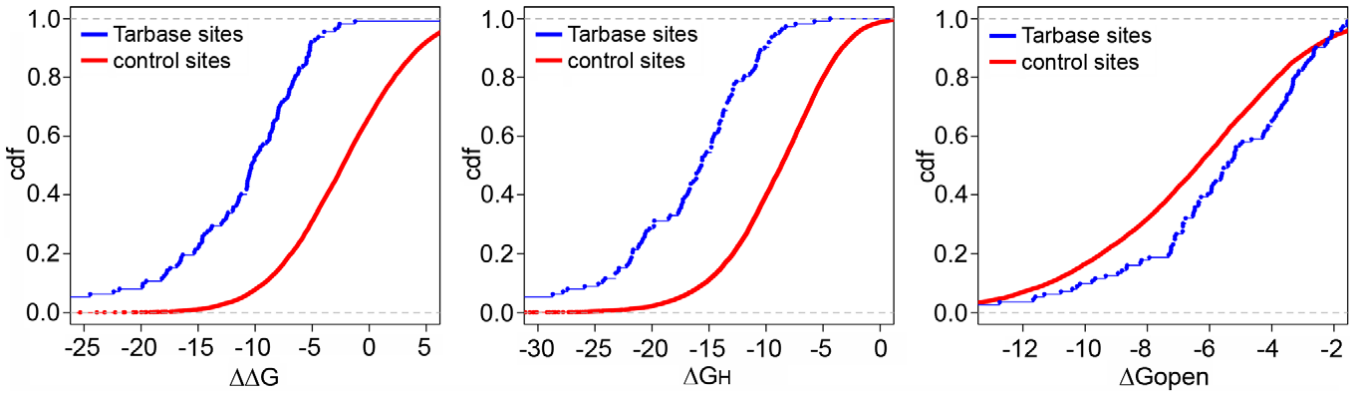
Cumulative density distribution of energy values at known miRNA target sites (Tarbase) vs. control sites. All energy values are in kcal/mol unit. (A) ΔΔG. (B) ΔG_H_. (C) ΔG_open_.

**Figure 2:**
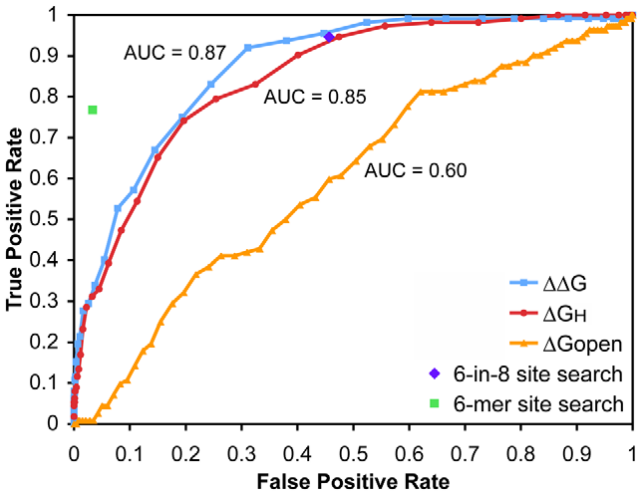
Contribution of energy values at Tarbase sites vs control sites. The plot shows ROC curves and corresponding AUC values for the three energy types in the duplex formation steps: ΔΔG, ΔG_H_, and ΔG_open_. The result for a canonical 6-mer seed match search and a search for relaxed seeds (at least 6 out of 8 positions—a 6-in-8 filter) are also shown.

We compared the total free energy change, the hybridization energy, and the disruption energy at reported true sites to the energy values at the control sites. The energy distributions significantly differed between the Tarbase sites and the control sites for all three energy types (p<1×10^−16^, p<1×10^−16^, and p = 4.3×10^−4^ based on Wilcoxon rank sum tests for ΔΔG, ΔG_H_, and ΔG_open_ respectively; Figure 1). The energy distributions also exhibited the correct shifts towards favorable duplex formations in true sites vs. control sites: lower ΔΔG and ΔG_H_, and higher ΔG_open_. To assess classification success between true and control sites, we used receiver operating characteristic (ROC) curves, which show true versus false positive rates at varying energy value cutoffs, and as well as the area under the curve (AUC); an AUC value of 1 indicates perfect classification, and a value of 0.5 indicates random performance. This analysis resulted in AUC values equaling 0.87, 0.85, and 0.60 for ΔΔG, ΔG_H_, and ΔG_open_ respectively (Figure 2). While this result supported the model that both steps of the duplex formation are important for target determination, hybridization energy was much more indicative on this set than mRNA accessibility. Comparing this strategy to a simple search for different seed types, Figure 2 shows that energy-based features improved upon simply looking for (imperfect) seed matches. For instance, our approach performed as well as scanning for a 6-in-8 (or better) match, while relying on the much less stringent requirement of a 4-mer match. Even though simple scans for 6-mer matches came with a greatly reduced false positive rate, they missed a considerable fraction of annotated sites. Our method, on the other hand, was able to eventually predict all positive Tarbase sites, not all of which contained canonical seed matches. Moreover, energy-based scoring consistently led to additional improvements when limiting the scoring to more stringent subsets of sites with 6-in-8 or canonical 6-mer seed matches (Figure S2A–B).

### Transcriptome-wide AGO protein occupation profiles exhibit preferable energy features

**Figure 3:**
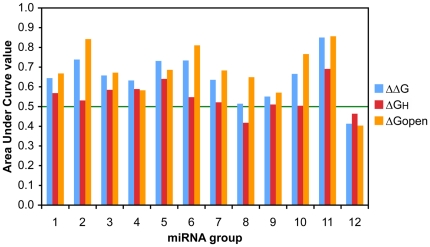
Discrimination between sites with and without evidence of AGO interaction. AUC values are shown for the three energy types in the duplex formation steps: ΔΔG, ΔG_H_, and ΔG_open_ for each miRNA group containing one or more of the 20 most highly expressed miRNAs in the same cell line and the miRNAs that shared their 5′end sequences. Only 6-mer sites were considered. The line at the AUC value of 0.5 indicates random performance.

We next investigated the energy contribution in distinguishing target sites at a genome-wide level. Recent studies have determined cross-linked sites of mRNA and AGO proteins, members of the RISC that shuttle miRNAs to their target sites [6]. Such data allow us to compare the energy values between seed matches in regions that interact with AGO proteins and seed matches in regions that show no interaction. While the site of interaction is mapped, the particular miRNA that is a part of the bound complex is generally unknown. We used the top 20 highly expressed miRNAs and clustered them with additional miRNAs that shared the same sequence at the 5′end. We computed energy values at seed matches (length 6 or longer) to the miRNA in these 12 non-redundant miRNA groups. Positive sites were matches within the cross-linked centered regions (CCRs) that contained one match location to seed sequences of our miRNA set. Negative sites were matches that lie outside any CCRs but fell into 3′UTRs of genes that were expressed in the same cell line. The AUC values showed a positive contribution of target site accessibility to distinguish AGO-interacting sites for all but one miRNA group, and strong contributions were observed in some (Figure 3). In contrast, signals from hybridization energy were not as strong as those observed in the Tarbase set.

These observed differences agree with the notion that these locations directly interact with AGO, yet lack direct evidence of which miRNA was involved. Our informed guess to assign specific miRNAs to CCRs may not always be accurate, and likewise, the experiment itself may not pick up all interacting sites, which means our negative set may still contain some false negatives. An important difference between the two datasets is that Tarbase contains experimentally supported target sites, but is certainly biased by early studies that generally assumed perfect seed matches, i.e. it can be expected to contain sites with strong hybridization. In turn, PAR-CLIP data demonstrate evidence of AGO binding, but not all of the interactions are stable or define functional sites, which agrees with comparatively lower hybridization energy values.

### Energy-based features are significantly correlated with miRNA-induced expression change

Having established the positive contribution of energy features at the site level, we investigated how such features would be correlated with genome-wide expression change at the gene level. Data on miRNA-induced changes at both mRNA and protein levels provided an appropriate framework to address this question [31], [32], and allowed us to compare the impact of miRNAs on gene expression at the transcriptional and translational level. Differently from evaluations of single sites, we will frequently observe multiple match locations in a 3′UTR. Therefore, we evaluated all 4-mer match sites in the 3′UTR, and evaluated correlations based on the top energy values and the sum over all sites. To avoid issues that may arise from the use of different technologies and experimental protocols, we here focused on data from one study, which used overexpression of individual miRNAs and assessed subsequent changes on the mRNA and protein levels by microarrays and pSILAC respectively [32].

We first assessed the correlation between individual energy features and observed expression change, using data at both mRNA and protein levels of five assayed human miRNAs (miR-1, miR-16, miR-30a, miR-155, and let-7b). Here, only miRNA-gene pairs with observed negative log_2_ fold change were used, as positive values were likely to result from indirect effects. Obviously, not all genes with an observed negative log_2_ fold change will be direct targets, and as a result, not all of them will contain miRNA target sites. In addition, less pronounced expression change may simply be a result of experimental error or precision. Out of these reasons, we did not expect to achieve high correlation values on this noisy set. Favorable duplex formation energy (lower ΔΔG, lower ΔG_H_, or higher ΔG_open_) should lead to positive correlation coefficients for ΔΔG and ΔG_H_, and a negative coefficient for ΔG_open_. For all three energy types, we investigated the correlation for top values of each energy type separately as well as the energy sum over all sites in order to take into consideration the frequent observation of multiple target sites of the same miRNA in a UTR. In addition, we included 3′UTR length in our feature set, as a chance for occurrence of target sites increases in a longer UTR; here a negative correlation to log_2_ fold change is expected. The results showed significant correlations between observed expression change and feature values (Table 1). For all of the features, the correlation was computed separately for the microarray (mRNA) and pSILAC (protein) datasets, and the correlation was generally stronger at the mRNA level than at the protein level. These significant correlations demonstrated that energy features are correlated with the outcome mediated by miRNAs.

**Table 1:** Correlation between energy features, as well as 3′UTR length, and observed level of down-regulation.

	mRNA		Protein	
Feature	corr. coef.	p-value	corr. coef.	p-value
best ΔΔG	0.182	<1×10^−16^	0.109	<1×10^−16^
second best ΔΔG	0.186	<1×10^−16^	0.115	<1×10^−16^
best ΔG_H_	0.207	<1×10^−16^	0.097	2×10^−15^
second best ΔG_H_	0.218	<1×10^−16^	0.110	<1×10^−16^
best ΔG_open_	−0.028	0.022	−0.074	1.4×10^−9^
second best ΔG_open_	−0.028	0.023	−0.093	1.9×10^−13^
ΔG_H_ at the best ΔΔG site	0.186	<1×10^−16^	0.092	5.6×10^−14^
ΔG_H_ at the second best ΔΔG site	0.198	<1×10^−16^	0.104	<1×10^−16^
ΔG_open_ at the best ΔΔG site	−0.005	0.67	−0.028	0.023
ΔG_open_ at the second best ΔΔG site	0.016	0.21	−0.019	0.14
Sum of ΔΔG	0.225	<1×10^−16^	0.094	1.5×10^−14^
Sum of ΔG_H_	0.185	<1×10^−16^	0.155	<1×10^−16^
Sum of ΔG_open_	0.139	<1×10^−16^	0.149	<1×10^−16^
3′UTR length	−0.138	<1×10^−16^	−0.133	<1×10^−16^

Shown are the Spearman correlation coefficient and the corresponding p-value between different individual energy or 3′UTR length, and the observed log_2_ fold change at the mRNA or protein level.

To assess whether a simple model combining these features could successfully predict the outcome on unseen data, we used the combination of energy features as well as 3′UTR length in a linear regression model to predict expression change. In a cross-validation setting, we pooled expression data from all experiments and divided the transcripts into five disjoint training and test sets, each of which contained expression data from all miRNAs. We built and tested linear models on mRNA and protein expression datasets separately in order to assess any differences on the mRNA or protein level. All features listed in Table 1 were used for model training. Given the large training dataset and the redundancy between some of the features, we consistently observed zero weights for several features: ΔGopen at the top two ΔΔG sites, and sum of ΔΔG. Spearman's correlation test was used to evaluate the model, and showed that the model was able to predict expression change with a significant correlation to the observed change at both mRNA and protein levels (Table 2); the correlation of the model combining multiple features was higher than the individual feature correlations in Table 1. Looking at the seed type components of putative targets defined at different observed expression changes, we found that the energy-based model was able to capture canonical sites without having to impose stringent seed rules, yet at the same time successfully identified highly down-regulated genes that lacked canonical match sites (Figure S3; see Results S1 for details).

**Table 2:** Correlation of linear model scores with observed level of down-regulation.

	mRNA		protein	
	Average corr. coef.	p-value	Average corr. coef.	p-value
5 miRNAs	0.241	<7×10^−15^	0.152	<5×10^−6^
4 miRNAs (excluding let-7b)	0.292	<2×10^−16^	0.185	<5×10^−8^

Shown is the average Spearman correlation coefficient between model-predicted score and observed log_2_ fold change, averaged across five cross-validation runs. The specified p-value is the upper bound among all five cross-validation runs. The model was trained and tested separately on expression change data at mRNA and protein level.

Upon closer investigation, we noted that the experiment for let-7b overexpression constituted an outlier, and cross-validation model performance on only the remaining 4 miRNA datasets showed marked improvement (Table 2). This corroborated the previous observation that, different from all of the other 4 miRNAs, the let-7b seed was not the most enriched sequence motif in the mRNA dataset [41]. One possible explanation may lie in the sequence composition of the miRNAs: unlike the other 4 miRNAs, hsa-let-7b consists of predominantly G and U bases (19 out of 22 bases), which may allow for more extensive G-U pairing and consequently for a more extensive set of imperfect and less effective target sites.

### A simple energy-based model identifies highly down-regulated targets, including imperfect sites

The study by Selbach et al [32] evaluated several miRNA target prediction tools, where targets were defined by protein level changes; the target prediction tools assessed in the study had typically been trained on mRNA data, as their study was among the first to measure the impact of miRNAs on protein synthesis. To align with this setup, and to assess the performance of our method for de novo target prediction, we used the model trained on mRNA expression change data to compute a prediction score, and used the observed protein log_2_ fold change to define bona fide targets. Since the genes with small negative log_2_ fold change were more likely to be noise or come from experimental errors, we retrained the model on only genes meeting an observed mRNA expression change cutoff of less than or equal to −0.1, and used the dataset excluding let-7b (see Results S1 and Figure S4 for results when all 5 miRNAs were used). To increase the stability of estimates, we used each model from the 5-fold cross validation to compute predicted scores, and used the median value as prediction for all genes, including genes with observed positive expression change.

**Figure 4:**
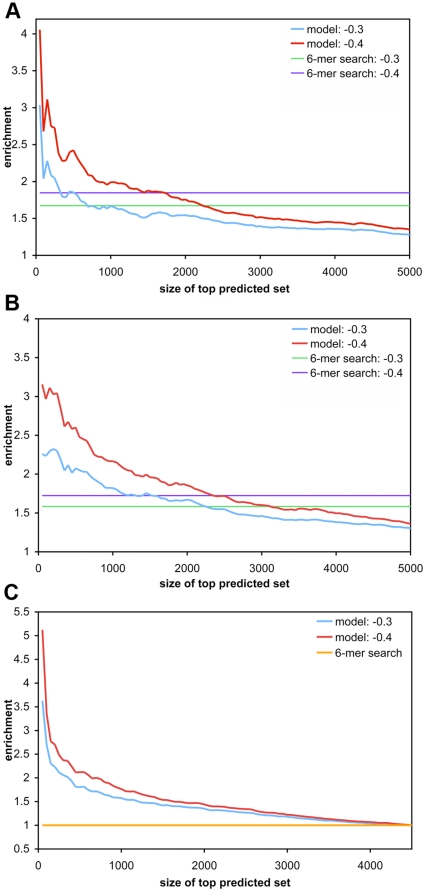
Enrichment of bona fide targets within top predicted target sets of varying size. Genes were ranked by median predicted score computed from five cross-validation models trained on mRNA expression data of four miRNAs (i.e. excluding let-7b) from Selbach et al. [32]. Bona fide targets were defined as those with observed protein log_2_ fold change less than or equal to different cutoffs, as observed in independent experiments by pSILAC; genes above the cutoff were considered as non-targets. The enrichment of bona fide targets in ranked sets of increasing size is shown. (A) all 4-mer sites were used in model training and predicted score computation. The plots for −0.3 and −0.4 log_2_ fold change cutoffs are shown. The corresponding baseline enrichment from a canonical 6-mer seed match search is also shown. (B) A 6-in-8 filter was applied to select for sites to compute feature values, and only 3′UTRs that have at least one 6-in-8 (or better) match were used in model training, and enrichment evaluation. (C) Similar to (B), but a filter for a 6-mer (or better) site was used. On this set, canonical 6-mer baseline corresponded to an enrichment value of 1.

To benchmark the model performance against random expectation, we computed the enrichment of highly down-regulated genes in top predicted gene sets (as ranked by the model score), compared to the full set. By varying the size of the top predicted set, instead of defining one threshold for target prediction, we were able to assess the trend of bona fide target enrichment across the full range of model scores. It was evident that the simple energy-based model was able to identify highly down-regulated genes as targets (Figure 4): there was a clear enrichment compared to random (an enrichment value of 1) with stronger enrichments for more negative prediction scores (smaller top predicted sets). We used different cutoffs to define bona fide targets, and enrichments were higher at more stringent cutoffs, indicating a favorable trend of our model to predict more of the genes with stronger observed down-regulation. Our method yielded stronger enrichments over a canonical 6-mer baseline even when all 4-mer matches were included (Figure 4A). While the enrichment decreased for the 6-in-8 set, the signal above baseline, a more suitable way to compare across the sets, became greater than the 4-mer set (Figure 4B). And with a more restricted site filter, the differences from 6-mer search baseline increased even more, and the enrichment value went up to five folds (Figure 4C).

Up to this point, our analyses did not directly address the important question whether the signals mainly came from perfect sites in the UTR, and whether imperfect sites actually contributed to the model performance. To determine the contribution of imperfect sites, we repeated training and evaluation of the prediction model on genes with only imperfect sites in the 3′UTR. Our gene set here was limited to 3′UTRs that do not have a perfect match of length 6 or longer, and contain only 6-in-8 or 7-in-8 sites. Figure S5A–B show that our method was able to predict enriched bona fide targets, and confirmed the enrichment trends of top predictions on UTRs that only have imperfect sites.

### Performance of the energy-based prediction model exceeds PITA algorithm

Previous studies reported that the performance of PITA, an energy-based miRNA target prediction approach that also uses energy in duplex formation, did not exceed a simple seed match search on mammalian data [31], [32]. Our energy-based model, on the contrary, yielded a stronger signal than a baseline 6-mer search. To investigate this, we directly compared the performance of our method to PITA [9]. Since PITA prediction requires canonical sites of length 6 or longer, we used a prediction model that was built on UTRs with at least one 6-mer (or longer) match to the seed sequence of the 4 miRNAs (i.e. model used for Figure 4C). In the subset of genes that overlap between our dataset and PITA predictions, we compared the enrichment of highly down-regulated genes as ranked by our predicted score to the enrichment when ranked by PITA score.

**Figure 5:**
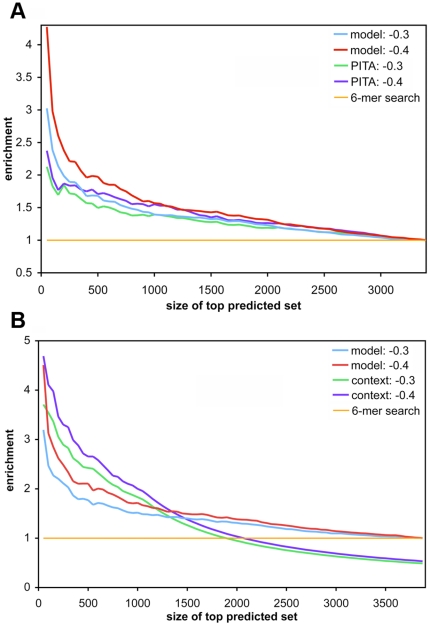
Comparison of the prediction model to other existing tools. (A) Our model score vs PITA score. Model-predicted scores and enrichment values were computed as in Figure 4C. We used PITA scores with flanking length of 3 and 15 bases upstream and downstream, for which the original study reported better performance compared to no flanking region [9]. (B) Our model score vs TargetScan's context score. Models from the 6-mer training set were used (cf. Figure 4C). TargetScan UTRs without the required canonical sites (i.e. no context score) were ranked at the bottom of the predicted list.

Our energy-based method yielded a higher enrichment than PITA score throughout the ranked predicted set (Figure 5A). The enrichment of bona fide targets on the common subset was again more pronounced for our top scores. Notably, PITA performance greatly decreased and became more uniform throughout the ranked list when let-7b was included (Figure S6A). Our method, on the other hand, showed a consistent performance on the 6-mer set with or without let-7b in model training and enrichment computation. This suggests that our method is more robust than PITA, and may explain the low performance of PITA observed in previous studies.

In conclusion, a model based solely on energy features and UTR length can deliver a favorable performance on mammalian data.

### Comparison to TargetScan context scores

Other than energy-based predictors, most target prediction tools rely on conservation and were thus not appropriate to compare against. Context scores of the popular TargetScan predictor, on the other hand, can be selected to ignore the contribution of conservation [14]. Similar to the comparison with PITA, we used the 6-mer prediction models to compute predicted scores for UTRs that have at least one 6-mer (or better) site. The comparison was limited to genes that are in both the TargetScan UTR sequence database and our UTR set. Both context score and our energy-based scoring sets yielded enriched bona fide targets in top predicted sets, and the enrichment was higher towards the higher-scoring gene sets (Figure 5B, S6B). Since TargetScan requires predicted targets to have canonical match sites of at least length 7, and we accordingly ranked genes that lacked 7-mer or 8-mer matches at the bottom of the context score predictions, it is not unexpected that context scores had higher enrichments in the top scoring sets, which were all based on longer seed matches. The context score performance eventually fell below our method, likely because it failed to identify bona fide targets that lack those long canonical sites in the 3′UTR. To evaluate this in more detail, we separately computed enrichment plots of genes with context scores vs. those without. This showed that the performance of our model fell below TargetScan on the subset of genes with context scores (i.e. genes containing canonical sites of length 7 or 8; Figure S7A–B), but delivered significant predictions of highly down-regulated genes that lack canonical seed matches, i.e. of putative targets that TargetScan did not score at all (Figure S7C–D). The comparison between our energy-based method and TargetScan's context score therefore ended in a tie – for long seeds, the additional features in the TargetScan model improved performance, but it missed bona fide non-canonical targets that our method was able to predict.

### Energy-based prediction yields enrichments of highly down-regulated genes on an independent dataset

**Figure 6:**
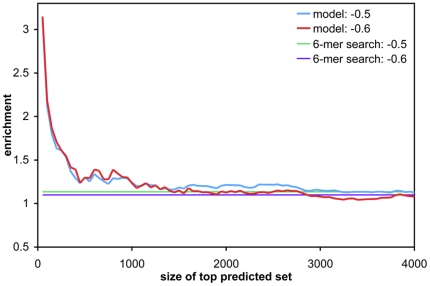
Enrichment of highly down-regulated mRNAs for miR-K12-11 target predictions. As in Figure 4, the enrichment of bona fide targets within gene sets of increasing size, ordered by increasing model score, is shown. A 6-in-8 filter was used for model training and prediction (cf. Figure 4B). Different observed mRNA log_2_ fold change values (−0.5, and −0.6) were used as a cutoff for bona fide targets. The corresponding baseline enrichments resulting from 6-mer seed matches are also shown.

In order to allow for a controlled assessment of energy-based target prediction, results so far were obtained on data from the same study. To conclude, we assessed the performance of the model on a dataset of human mRNA expression changes induced by miR-K12-11. This miRNA is encoded by Kaposi's sarcoma-associated herpesvirus (KSHV), and has been shown to be a functional ortholog to a human miRNA, miR-155 [22]. Given that model trained on the 6-in-8 set yielded high enrichments above baseline while including predictions of imperfect sites (Figure 4B), we used the model trained on 3′UTRs with at least one 6-in-8 (or better) site, and restricted evaluation to sites with at least a minimal 6-in-8 seed. As in the previous enrichment analyses, we used the models from the five-fold cross-validation training on mRNA expression change data from Selbach et al. [32] above to compute predicted expression change caused by miR-K12-11, and used the median value to compute the enrichment values. Figure 6 shows the enrichment of down-regulated genes below an observed mRNA log_2_ fold change cutoff in our top predicted sets (cf. Figure S8 for results on a model trained on all 5 miRNAs). Compared to the enrichment analyses in the previous section, the enrichment here was constrained to a smaller top set of genes. This difference was likely due to different experimental setups: unlike in the overexpression studies in Selbach et al. [32], the viral miRNA was transfected at physiological levels, and the lower overall number of potential targets agrees with fewer genes showing significant expression changes in the viral dataset. Nevertheless, the enrichment from our model was again higher than a search for 6-mer sites in the 3′UTRs, thus clearly improving on the standard baseline approach when conservation across target sites cannot be used as feature, while at the same time allowing for mismatches in the target site.

## Discussion

The main intent of this study was to evaluate the utility of energy-based models for miRNA target prediction, which had initially shown promise to provide a framework to handle the prediction of non-conserved targets, and/or targets with imperfect seed matches. After initial reports that approaches such as PITA might outperform “classical” target predictors [9], follow-up studies reported contrary results, suggesting that some energy-based methods might not even exceed baseline results from simple scanning for canonical seed matches, at least for mammalian genes [31], [32], [42]. Here, we were able to reconcile these conflicting results: while an evaluation of PITA predictions showed a mixed performance and was greatly affected by miRNAs in the set, our simple linear regression model based on energy features was able to predict genes down-regulated by miRNAs with clear enrichments.

The evaluation of individual target sites included both small manually curated sets, and genomic dataset from recent PAR-CLIP experiments that identified binding location of AGO proteins. The results reflected strong biases in either set: experimentally supported mammalian mRNA targets in Tarbase were largely defined by mRNA:miRNA hybridization energy, and provided a noticeable but modest difference in local secondary structure. In contrast, genome-wide energy evaluations at the site level suggested a stronger impact of accessibility on AGO-interacting sites. Here, hybridization energy contributed noticeably less, hinting at the difficulty in pinpointing the precise miRNA acting at the target location, and/or at an overall lower effect of hybridization on sites that were occupied yet whose functional effect on expression levels is unknown. These findings underlined some of the global statistics reported in the original study [6]: in their study, seed matches to the top 100 expressed miRNAs were significantly enriched in CCRs, but at a relatively low level of 1.5-fold when compared to randomized 6-mers. However, CCRs were overall found to be located in region of increased accessibility.

Evaluations on genomic mRNA and protein expression change data at the UTR level confirmed contributions of both energy components in the duplex formation steps. The stronger correlation to mRNA expression change, compared to protein synthesis change, of a simple linear model agrees with a recent report which suggested changes in mRNA levels as the predominant effect of miRNA-induced gene regulation [43]. Our results indicated that commonly used hierarchies of seed matches are naturally reflected in energy-based scores (cf. Results S1). As such, stronger expression change in the presence of longer seed matches also means a more favorable energy score at these sites, which provides for an intuitive and natural way to incorporate seed mismatches and different seed types in the prediction model. While restrictions to canonical seed matches provide for a higher enrichment of true targets in predictions, our energy-based method was able to predict non-canonical targets even without using conservation. As we demonstrated on the dataset of viral miRNA induction, the current approach is best used to define putative targets when given functional genomics data: rank genes by prediction scores, determine an enrichment profile based on a reasonable cutoff for significant expression change, and investigate (non-canonical) putative targets in a suitable set of top predictions.

Re-assessing the potential of energy-based features and models for target prediction, we provided convincing evidence that such models can indeed deliver promising results and naturally include imperfect sites. In particular, evaluations at both site and UTR levels demonstrated the usefulness of thermodynamics features for miRNA target identification in mammals at the genome-wide level, and not just in model organisms with shorter 3′UTRs which had been the main focus in other studies of energy-based target identification [8], [9]. Our results showed that genes with stronger down-regulation were enriched in the top predictions, and our method yielded consistent favorable performance in comparison against other tools, and in target prediction on an independent dataset. While our current approach is already competitive for the prediction of non-canonical sites, a more stringent training on clearly defined positive and negative targets would likely improve the performance, and future investigation on possible effects of differences among miRNA sequences could help improve robustness of the tool on a relaxed seed match dataset.

It will soon be possible to intersect functional genomics datasets assessing the impact of miRNAs on transcript and protein levels with the increasingly available CLIP data, which define putative target sites at a genomic scale. This will allow for defining effective training sets, whose absence has hindered the prediction of targets. Additionally, combining energy with conservation scores or sequence features such as mRNA local composition or relative position of target sites is likely to prove informative [44]. In summary, energy-based models provide a natural and promising starting point, and deserve a renewed attention for more comprehensive modeling efforts to predict microRNA targets.

## Supporting Information

Figure S1 **ΔΔG values and normalized luciferase ratios for several** ***Drosophila*** **miRNAs and their targets in different UTR context.** The ΔΔG values (in kCal/mol) were computed as described in the Methods section. The normalized luciferase ratios were obtained from the original study [9]. Spearman correlation test was used to compute the correlation coefficient.
(TIF)

Figure S2 **ROC plot of energy values at Tarbase sites vs control sites with seed type filter.** The plot shows the three energy types in the duplex formation steps: ΔΔG, ΔG_H_, and ΔG_open_. (A) For both Tarbase and control sets, we used the same sites as in Figure 2, but with a 6-in-8 site filter. Thus, the positive set here corresponded to 95% of the full Tarbase set in Figure 2. (B) Same as in (A), but further restricting sites to canonical matches of at least 6 consecutive base pairs (77% of the Tarbase sites in Figure 2).
(TIF)

Figure S3 **Seed type composition of gene sets down-regulated by miRNA overexpression.** Genes assayed in a miRNA overexpression study were ranked by observed protein log_2_ fold change. The plots show the fraction of different seed types in the top down-regulated sets of an increasing size (50 genes increment). The seed type of the UTR was determined among seed type of all matches according to an order: 8-mer, 7-mer-A, 7-mer, 6-mer, 7-in-8, 6-in-8, 5-mer, and 5-in-8. The seed type symbols are as specified in Methods, including the remaining 4-mer sites that do not have any additional base pairs within an 8-mer region (4-mer). Only the genes with negative observed log_2_ protein fold change were included in the plots. As reference, the asterisk marks the size of the top down-regulated gene set that corresponds to an observed protein log_2_ fold change less than or equal to −0.2. Note that the 7-mer-A seed type has no counts here since all five miRNAs have U at position 1, which means the 7-mer-A type is the same as the 8-mer type in this case, and our seed type order as a result assigns such site/UTR as an 8-mer. (A) Composition of UTR seed type of all genes with observed down-regulation at the protein level. (B) Composition of UTR seed type for the subset of genes in (A) that were predicted as a target by the linear model. These putative targets were genes with predicted score less than a cutoff determined from cross-validation runs. (C) Same as in (B), but showing the seed type of the best ΔΔG site in each gene.
(TIF)

Figure S4 **Enrichment of bona fide targets within top predicted target sets for the 5-miRNA set.** Similar to Figure 4, but all five miRNAs were used to train the model and included in the enrichment analysis. (A) all 4-mer sites were used (B) with a 6-in-8 site filter (C) with a 6-mer site filter.
(TIF)

Figure S5 **Enrichment analysis on strictly imperfect UTRs.** Genes with only imperfect sites of at least length six in the 3′UTR were used for model training and enrichment analysis. (A) on the 4-miRNA set (B) on the 5-miRNA set.
(TIF)

Figure S6 **Comparison of the energy-based model to other existing tools for the 5 miRNA datasets.** Similar to Figure 5, but 5 miRNA datasets were used in model training and enrichment analysis. (A) Our model scores vs PITA scores. (B) Our model scores vs TargetScan's context scores.
(TIF)

Figure S7 **Additional comparison of the energy-based model to TargetScan's context score.** (A) Enrichment of highly down-regulated genes in top predicted set, ranked by context score vs. our model score on the subset of genes in Figure 5B that have reported context score. (B) Same as (A), but all 5 miRNAs were included in model training and enrichment calculation. (C) Predictions ranked by our model score on the genes that do not have context score (i.e. the complement of the gene set in (A)). (D) Same as (C), but for the 5-miRNA set.
(TIF)

Figure S8 **Enrichment of highly down-regulated mRNAs for miR-K12-11 target predictions using the models trained on 5 miRNAs.** Similar to Figure 6, but all 5 miRNA datasets were used to train the models.
(TIF)

Table S1 **miRNA groups in the evaluation on PAR-CLIP dataset.**
(DOC)

Results S1 (DOC)
