# Supplementary figures and images for: Assessing the Utility of Thermodynamic Features for microRNA Target Prediction under Relaxed Seed and No Conservation Requirements
Source: PLoS One. 2011 Jun 6;6(6):e20622. doi: 10.1371/journal.pone.0020622 (PMC3108951; doi:10.1371/journal.pone.0020622)

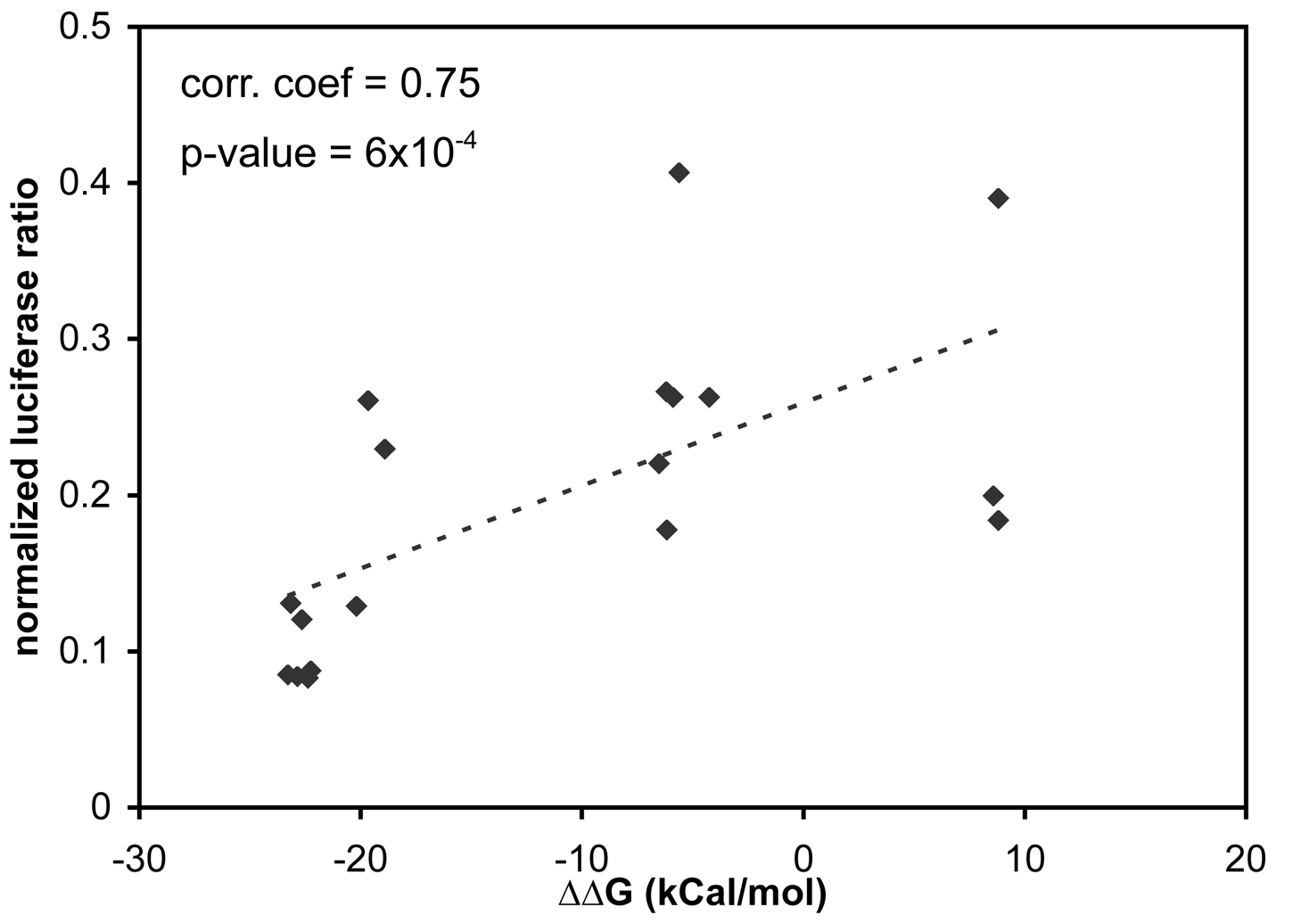

Supplement: Figure S1 — ΔΔG values and normalized luciferase ratios for several Drosophila miRNAs and their targets in different UTR context. The ΔΔG values (in kCal/mol) were computed as described in the Methods section. The normalized luciferase ratios were obtained from the original study [9]. Spearman correlation test was used to compute the correlation coefficient. (TIF) [file pone.0020622.s001.tif]

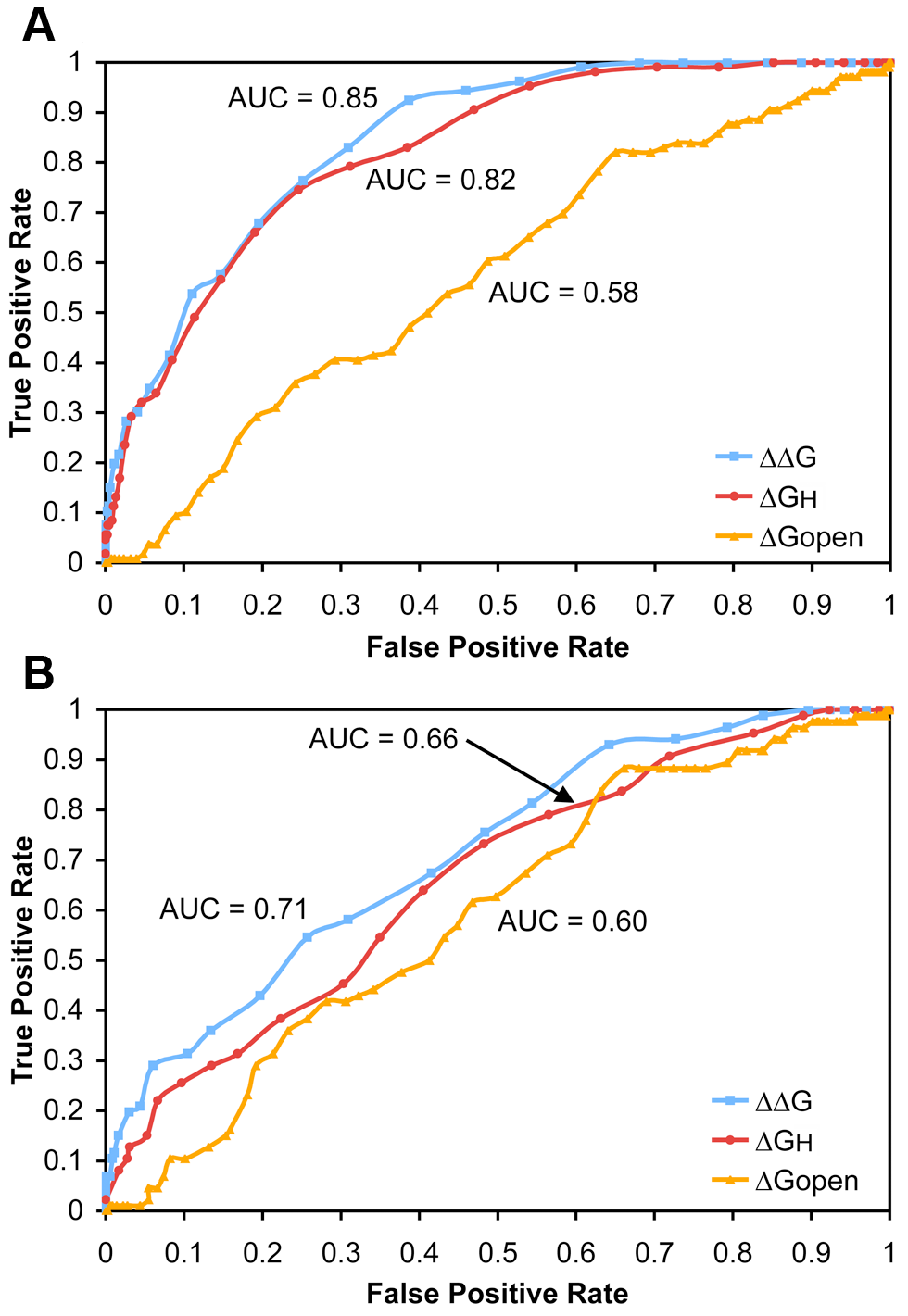

Supplement: Figure S2 — ROC plot of energy values at Tarbase sites vs control sites with seed type filter. The plot shows the three energy types in the duplex formation steps: ΔΔG, ΔGH, and ΔGopen. (A) For both Tarbase and control sets, we used the same sites as in Figure 2, but with a 6-in-8 site filter. Thus, the positive set here corresponded to 95% of the full Tarbase set in Figure 2. (B) Same as in (A), but further restricting sites to canonical matches of at least 6 consecutive base pairs (77% of the Tarbase sites in Figure 2). (TIF) [file pone.0020622.s002.tif]

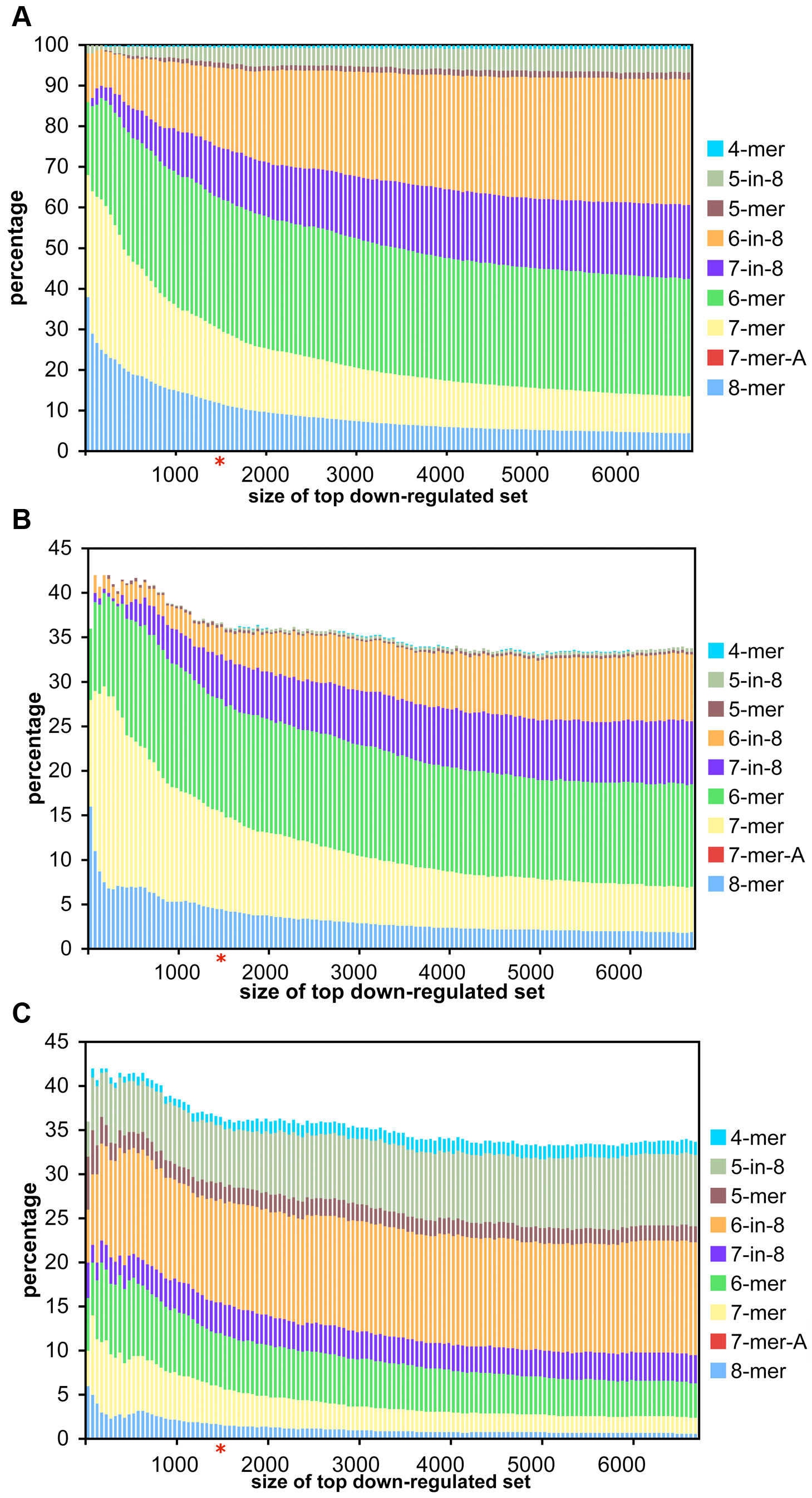

Supplement: Figure S3 — Seed type composition of gene sets down-regulated by miRNA overexpression. Genes assayed in a miRNA overexpression study were ranked by observed protein log2 fold change. The plots show the fraction of different seed types in the top down-regulated sets of an increasing size (50 genes increment). The seed type of the UTR was determined among seed type of all matches according to an order: 8-mer, 7-mer-A, 7-mer, 6-mer, 7-in-8, 6-in-8, 5-mer, and 5-in-8. The seed type symbols are as specified in Methods, including the remaining 4-mer sites that do not have any additional base pairs within an 8-mer region (4-mer). Only the genes with negative observed log2 protein fold change were included in the plots. As reference, the asterisk marks the size of the top down-regulated gene set that corresponds to an observed protein log2 fold change less than or equal to −0.2. Note that the 7-mer-A seed type has no counts here since all five miRNAs have U at position 1, which means the 7-mer-A type is the same as the 8-mer type in this case, and our seed type order as a result assigns such site/UTR as an 8-mer. (A) Composition of UTR seed type of all genes with observed down-regulation at the protein level. (B) Composition of UTR seed type for the subset of genes in (A) that were predicted as a target by the linear model. These putative targets were genes with predicted score less than a cutoff determined from cross-validation runs. (C) Same as in (B), but showing the seed type of the best ΔΔG site in each gene. (TIF) [file pone.0020622.s003.tif]

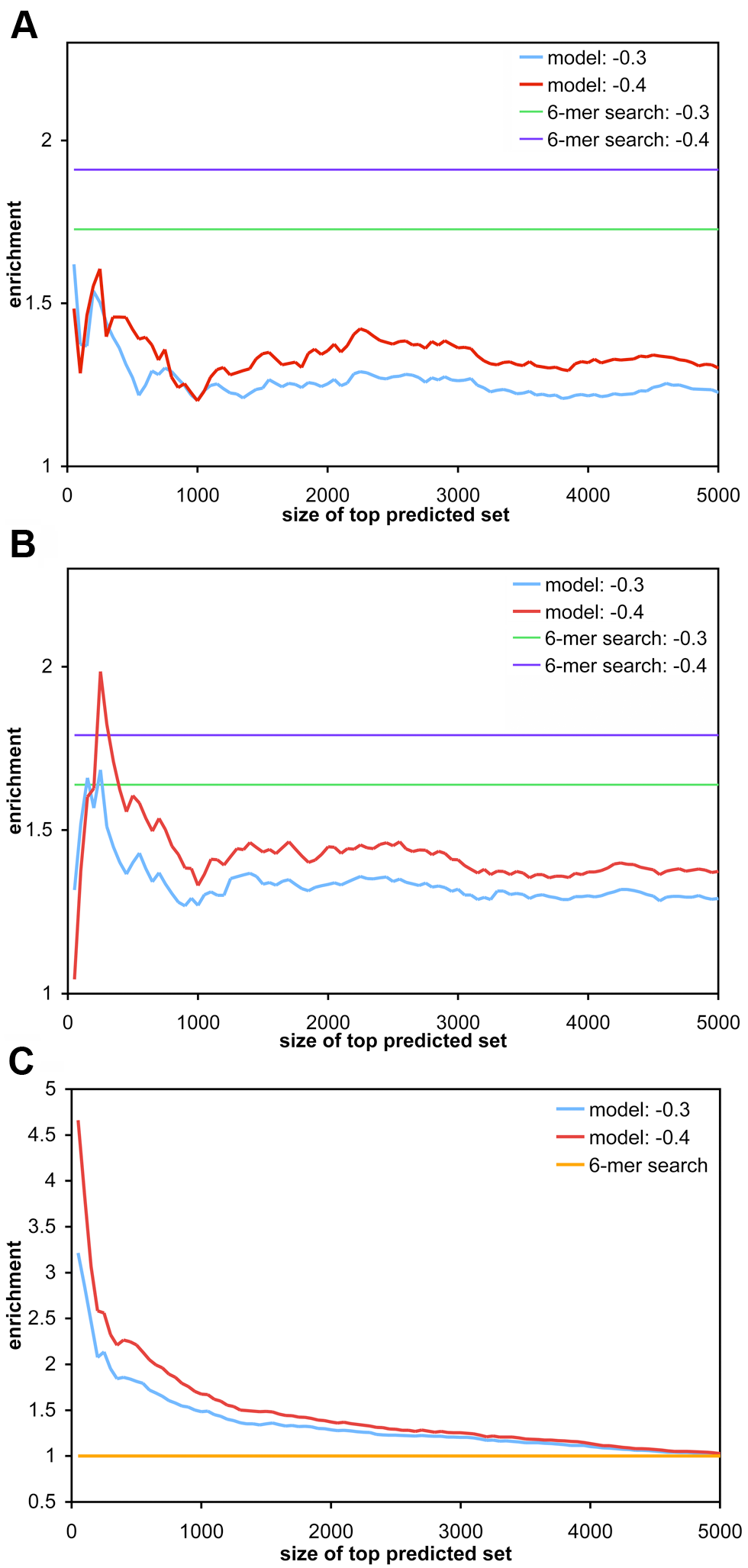

Supplement: Figure S4 — Enrichment of bona fide targets within top predicted target sets for the 5-miRNA set. Similar to Figure 4, but all five miRNAs were used to train the model and included in the enrichment analysis. (A) all 4-mer sites were used (B) with a 6-in-8 site filter (C) with a 6-mer site filter. (TIF) [file pone.0020622.s004.tif]

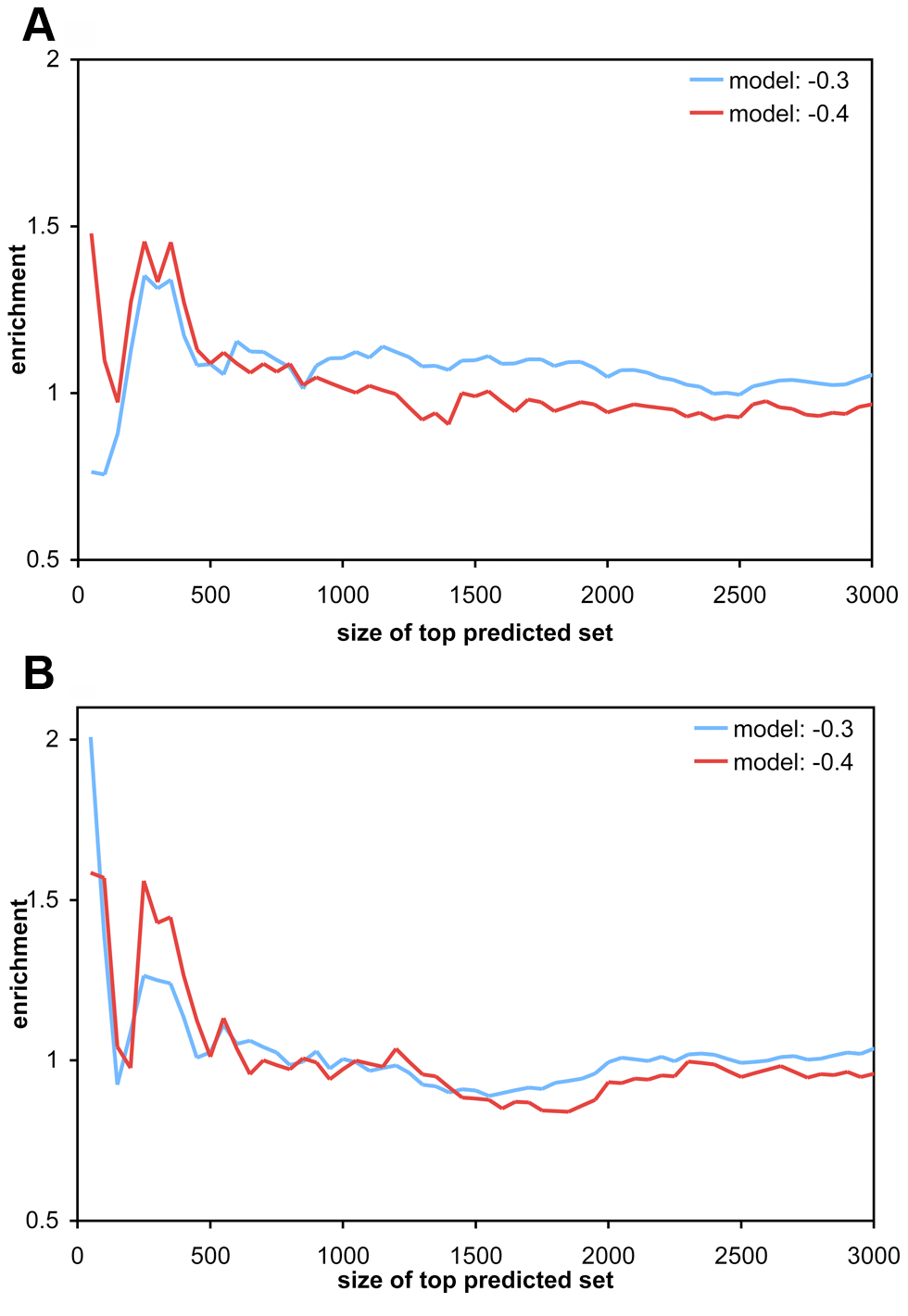

Supplement: Figure S5 — Enrichment analysis on strictly imperfect UTRs. Genes with only imperfect sites of at least length six in the 3′UTR were used for model training and enrichment analysis. (A) on the 4-miRNA set (B) on the 5-miRNA set. (TIF) [file pone.0020622.s005.tif]

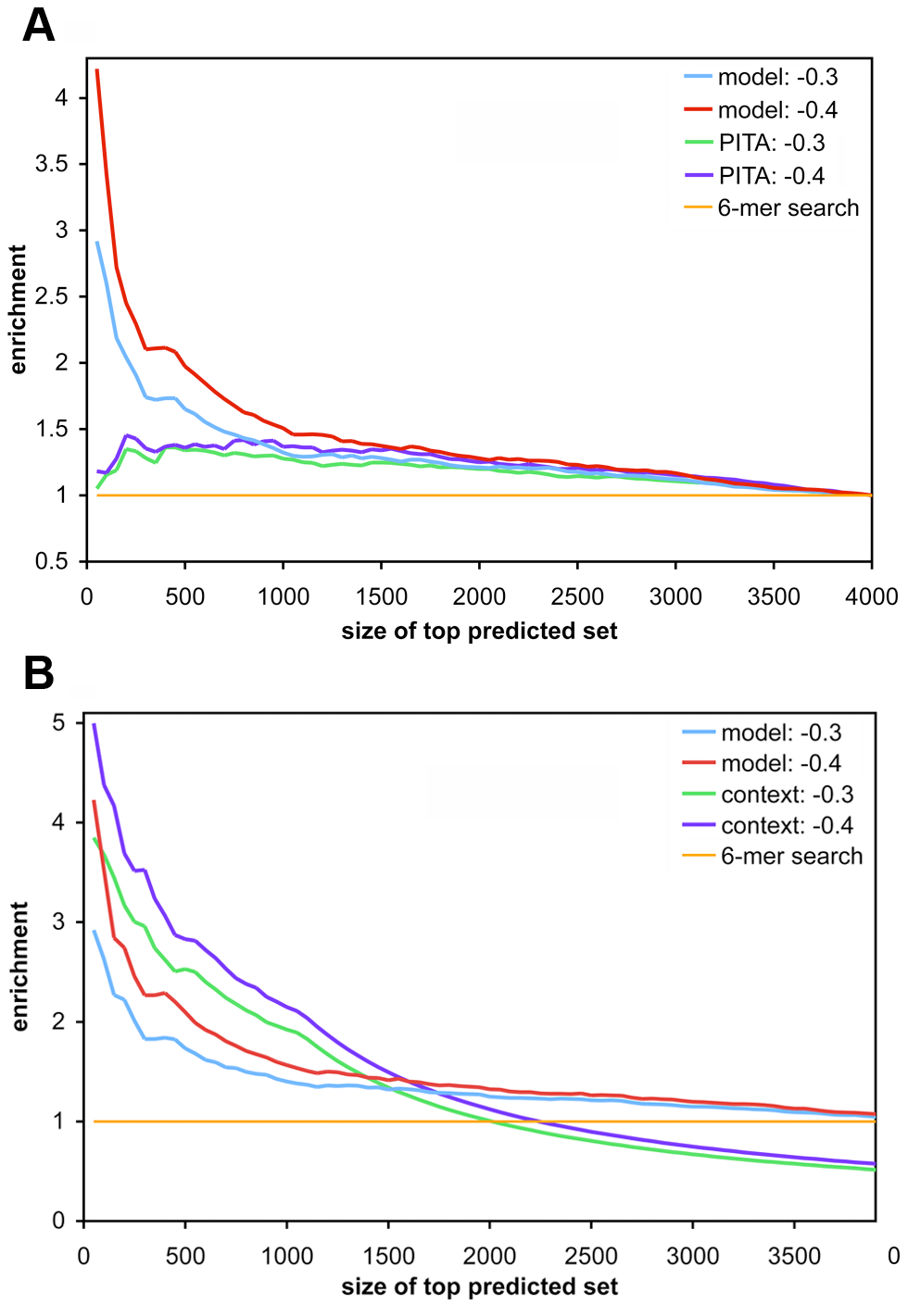

Supplement: Figure S6 — Comparison of the energy-based model to other existing tools for the 5 miRNA datasets. Similar to Figure 5, but 5 miRNA datasets were used in model training and enrichment analysis. (A) Our model scores vs PITA scores. (B) Our model scores vs TargetScan's context scores. (TIF) [file pone.0020622.s006.tif]

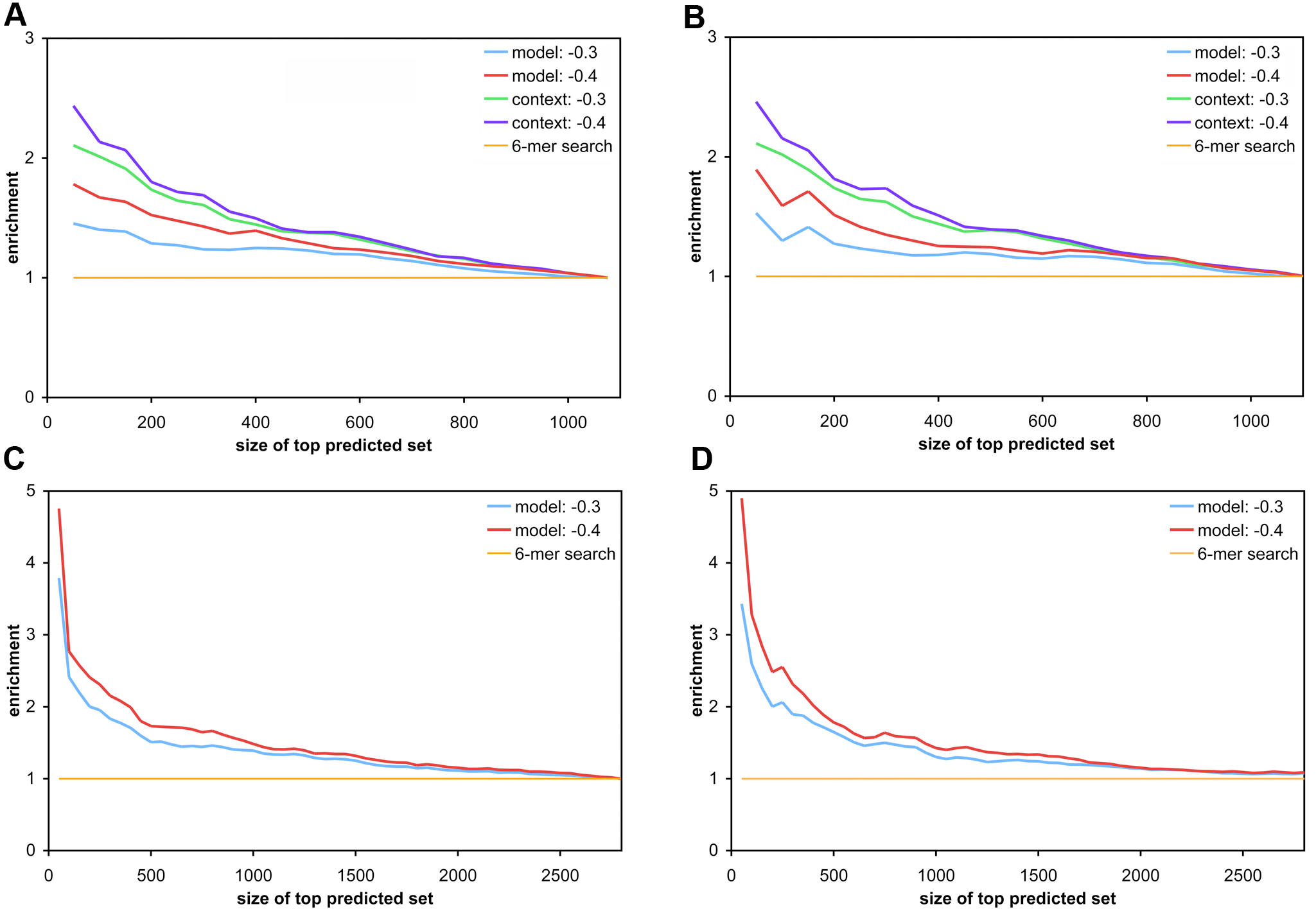

Supplement: Figure S7 — Additional comparison of the energy-based model to TargetScan's context score. (A) Enrichment of highly down-regulated genes in top predicted set, ranked by context score vs. our model score on the subset of genes in Figure 5B that have reported context score. (B) Same as (A), but all 5 miRNAs were included in model training and enrichment calculation. (C) Predictions ranked by our model score on the genes that do not have context score (i.e. the complement of the gene set in (A)). (D) Same as (C), but for the 5-miRNA set. (TIF) [file pone.0020622.s007.tif]

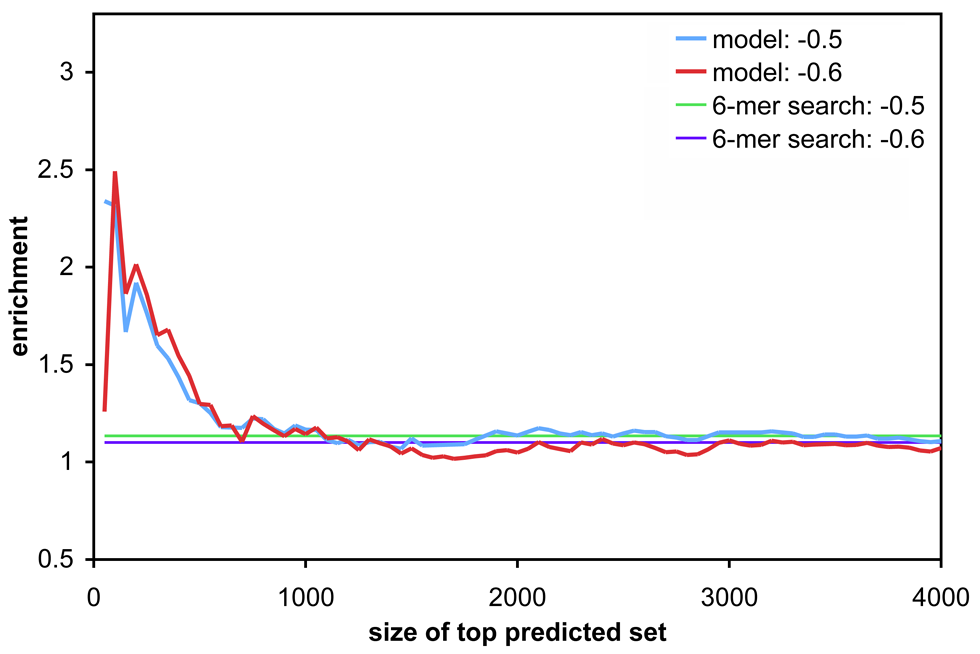

Supplement: Figure S8 — Enrichment of highly down-regulated mRNAs for miR-K12-11 target predictions using the models trained on 5 miRNAs. Similar to Figure 6, but all 5 miRNA datasets were used to train the models. (TIF) [file pone.0020622.s008.tif]
