# Supplementary material for: Assessing the Utility of Thermodynamic Features for microRNA Target Prediction under Relaxed Seed and No Conservation Requirements
Source: PLoS One. 2011 Jun 6;6(6):e20622. doi: 10.1371/journal.pone.0020622 (PMC3108951; doi:10.1371/journal.pone.0020622)
Supplement: Table S1 — miRNA groups in the evaluation on PAR-CLIP dataset. (DOC) [file pone.0020622.s009.doc]

**Table S1. miRNA groups in the evaluation on PAR-CLIP dataset.**

| group | representative miRNA | miRNAs |
| --- | --- | --- |
| 1 | hsa-miR-93 | hsa-miR-17, hsa-miR-20a, hsa-miR-20b, hsa-miR-93, hsa-miR-106a, hsa-miR-106b |
| 2 | hsa-miR-103 | hsa-miR-15a, hsa-miR-15b, hsa-miR-16, hsa-miR-103, hsa-miR-107, hsa-miR-195, hsa-miR-424, hsa-miR-497 |
| 3 | hsa-let-7a | hsa-let-7a, hsa-let-7b, hsa-let-7c, hsa-let-7d, hsa-let-7e, hsa-let-7f, hsa-let-7g, has-let-7i, hsa-miR-98 |
| 4 | hsa-miR-92a | hsa-miR-25, hsa-miR-32, hsa-miR-92a, hsa-miR-92b, hsa-miR-363 |
| 5 | hsa-miR-19b | hsa-miR-19a, hsa-miR-19b |
| 6 | hsa-miR-10a | hsa-miR-10a, hsa-miR-10b |
| 7 | hsa-miR-101 | hsa-miR-101 |
| 8 | hsa-miR-21 | hsa-miR-21 |
| 9 | hsa-miR-30e | hsa-miR-30a, hsa-miR-30b, hsa-miR-30c, hsa-miR-30d, hsa-miR-30e |
| 10 | hsa-miR-148a | hsa-miR-130a, hsa-miR-130b, hsa-miR-148a, hsa-miR-148b, hsa-miR-152, hsa-miR-301a, hsa-miR-301b |
| 11 | hsa-miR-18a | hsa-miR-18a, hsa-miR-18b |
| 12 | hsa-miR-186 | hsa-miR-186 |

Each group is represented by miRNA with the highest level of expression in HEK-293 cells among the members.
