# Supplementary material for: Assessing the Utility of Thermodynamic Features for microRNA Target Prediction under Relaxed Seed and No Conservation Requirements
Source: PLoS One. 2011 Jun 6;6(6):e20622. doi: 10.1371/journal.pone.0020622 (PMC3108951; doi:10.1371/journal.pone.0020622)
Supplement: Results S1 — (DOC) [file pone.0020622.s010.doc]

# *Supplementary Results*

**Seed type composition in predicted gene sets**

Given that energy-based approaches make minimal requirement on seed matches, it was instructive to evaluate which seed types were present in the Selbach et al dataset used in the main manuscript, and what fractions our energy-based predictions contained. Figure S3A shows the distribution of seed types across all down-regulated 3’UTRs, ranked by the observed level of protein down-regulation. As has been previously observed, stronger (more negative) log_2_ fold changes in this dataset were correlated with increased presence of canonical and longer seed matches. In the top 5-10% spectrum of down-regulated genes, about 80-85% of the 3’UTRs had at least one perfect 6-mer seed match. However, at lower but still prominent levels of expression change, the fraction of imperfect matches increased: at a log_2_ protein fold change cutoff of -0.2, more than a third of the 3’UTRs did not have a canonical Watson-Crick seed match of length 6 or longer.

To contrast seed match types in all genes with the seed types in well-scoring genes under the model, we determined a suitable cutoff for the model as fitted on mRNA expression changes (Table 2), and applied it to group genes into putative targets and non-targets. We determined the prediction score that led to the most significant p-value between observed expression change distribution of top predicted set and full set, and used the average of these values across the cross-validation sets as the cutoff. Comparing the presence of different seed types in the subset predicted by the model (Figure S3B) with the complete set (Figure S3A) confirmed that energy-based predictions were enriched in highly down-regulated genes. Furthermore, predictions for the most strongly down-regulated genes largely contain canonical seed matches in the 3’UTRs, with an enrichment of longer matches towards the top.

Even though the fraction of imperfect site matches was lower in the predicted set (Figure S3B) than in the total set (Figure S3A), about a quarter of predicted genes with an observed down-regulation of -0.2 or stronger contained only imperfect matches. Most of these concerned sites had one or two mismatches in the 8-nt seed region (7-in-8 and 6-in-8 seed type, respectively). Interestingly, when we looked at the sites that the model actually based the predictions on (i.e. lowest ∆∆G sites) (Figure S3C), rather than the mere presence in the UTR, it became apparent that the best energy scores were more frequently obtained at sites corresponding to imperfect seed matches. On the other hand, relatively few predictions were based on sites that passed only the minimal 4-nt match requirements, indicating that the liberal seed premise did not introduce extensive amounts of noise. Taken together, the energy-based target prediction agreed with previous approaches in the presence of longer seed matches at strong down-regulation, yet without the need of defining an extensive hierarchy of seed rules.

**Enrichment of bona fide targets on the full dataset (five miRNAs)**

Complementing the enrichment analysis on 4 miRNA datasets, we retrained the model with let-7b data included, and evaluated the performance on the 5 miRNA datasets. We observed bona fide target enrichments above random (i.e. enrichment greater than 1; Figure S4). When all 4-mer sites were included, our method’s enrichments did not exceed the baseline for a 6-mer search; however, when restricting the candidate sites for energy score computation to sites that passed a 6-in-8 site filter, we observed signal above baseline towards the top of the ranked list. With a 6-mer site filter, our method gained marked improvements: bona fide target enrichments based on our model score went up to 4.5 folds, and showed a clear signal above the 6-mer baseline. In fact, performance on the 6-mer set was comparable to that of the 4-miRNA set (Figure 4C). This 6-mer model also yielded superior performance than PITA (Figure S6A): our model score yielded similar enrichment trends compared to the 4-miRNA set (Figure 5A), while enrichment values and trends of PITA score were greatly affected when let-7b was included. The performance of the 6-mer model against TargetScan’s context score (Figure S6B) was also comparable to the 4-miRNA set (Figure 5B). These results suggested that despite possible effects of differences among miRNAs, our method with a 6-mer filter still yielded similarly favorable results. Further investigation may provide possible improvements by accounting for such differences when allowing for imperfect matches.
